# Symbiotic functioning and bradyrhizobial biodiversity of cowpea (*Vigna unguiculata *L. Walp.) in Africa

**DOI:** 10.1186/1471-2180-10-89

**Published:** 2010-03-23

**Authors:** Flora Pule-Meulenberg, Alphonsus K Belane, Tatiana Krasova-Wade, Felix D Dakora

**Affiliations:** 1 Department of Biotechnology, Tshwane University of Technology, Arcadia Campus, 175 Nelson Mandela Drive, Private Bag X680, Pretoria 0001, South Africa; 2 Department of Crop Science, 1 Stateartillery Road, Tshwane University of Technology, Pretoria Campus, Private Bag X680, Pretoria 0001, South Africa; 3 Department of Chemistry, Tshwane University of Technology, Arcadia Campus, 175 Nelson Mandela Drive, Private Bag X680, Pretoria 0001, South Africa; 4 Laboratoire Commun de Microbiologie (LCM) IRD/ISRA/UCAD, BP 1386, CP 18524, Dakar, Senegal

## Abstract

**Background:**

Cowpea is the most important food grain legume in Sub-Saharan Africa. However, no study has so far assessed rhizobial biodiversity and/or nodule functioning in relation to strain IGS types at the continent level. In this study, 9 cowpea genotypes were planted in field experiments in Botswana, South Africa and Ghana with the aim of i) trapping indigenous cowpea root-nodule bacteria (cowpea "rhizobia") in the 3 countries for isolation, molecular characterisation using PCR-RFLP analysis, and sequencing of the 16S - 23S rDNA IGS gene, ii) quantifying N-fixed in the cowpea genotypes using the ^15^N natural abundance technique, and iii) relating the levels of nodule functioning (i.e. N-fixed) to the IGS types found inside nodules.

**Results:**

Field measurements of N_2 _fixation revealed significant differences in plant growth, δ^15^N values, %Ndfa and amounts of N-fixed between and among the 9 cowpea genotypes in Ghana and South Africa. Following DNA analysis of 270 nodules from the 9 genotypes, 18 strain IGS types were found. Relating nodule function to the 18 IGS types revealed significant differences in IGS type N_2_-fixing efficiencies. Sequencing the 16S - 23S rDNA gene also revealed 4 clusters, with cluster 2 forming a distinct group that may be a new *Bradyrhizobium *species. Taken together, our data indicated greater biodiversity of cowpea bradyrhizobia in South Africa relative to Botswana and Ghana.

**Conclusions:**

We have shown that cowpea is strongly dependant on N_2 _fixation for its N nutrition in both South Africa and Ghana. Strain IGS type symbiotic efficiency was assessed for the first time in this study, and a positive correlation was discernible where there was sole nodule occupancy. The differences in IGS type diversity and symbiotic efficiency probably accounts for the genotype × environment interaction that makes it difficult to select superior genotypes for use across Africa. The root-nodule bacteria nodulating cowpea in this study all belonged to the genus *Bradyrhizobium*. Some strains from Southern Africa were phylogenetically very distinct, suggesting a new *Bradyrhizobium *species.

## Background

Cowpea (*Vigna unguiculata *L. Walp.) is a major food crop in Africa, where its leaves, green pods and grain are eaten as a dietary source of protein. The cowpea grain contains about 23% protein and 57% carbohydrate, while the leaves contain between 27 - 34% protein [1]. The leaves and grain are also supplied as high protein feed and fodder to livestock. Cowpea is the most commonly grown food legume by traditional farmers in Sub-Saharan Africa, possibly because of its relatively wide adaptation to drought and low-nutrient environments. Cowpea freely forms root nodules with some members of the Rhizobiaceae such as *Rhizobium *and *Bradyrhizobium *[2]. It is inside these nodules where nitrogenase enzyme in rhizobium bacteroids reduces N_2 _into NH_3 _via the GS/GOGAT pathway, leading to exchange of nitrogenous solutes with host plant for recently-formed photosynthate.

A survey of N_2 _fixation in farmers' fields showed that cowpea can derive up to 66% of its N from symbiotic fixation in Botswana [3], and up to 99% in Ghana [4]. The observed N contribution by this mutualistic relationship between cowpea and species of *Rhizobium *and *Bradyrhizobium *forms the basis for its importance in cropping systems. Moderate grain yields of 1500 kg/ha have been reported for cowpea in Ghana [1] and over 2600 kg/ha in South Africa [5]. To increase the threshold of cowpea yields in Africa would require identification of genotypes that exhibit high symbiotic performance and better plant growth.

Because cowpea nodulates freely with both rhizobia and bradyrhizobia [1], it is often described as being promiscuous. Yet only few studies [1,6-9] have examined the biodiversity of cowpea rhizobia and bradyrhizobia in Africa, the native home of this legume species. One study [6] reported four different *Bradyrhizobium *strains belonging to 3 genospecies, and concluded that the cowpea rhizobia appeared to be more diverse in arid areas. Recently, another study [8] grouped cowpea rhizobia from China into six genospecies, and linked microsymbiont distribution and diversity to geographical location.

Like most published reports on the biodiversity of root-nodule bacteria, namely rhizobia, bradyrhizobia, azorhizobia, sinorhizobia and mesorhizobia, none of the studies [1,6-9] on cowpea rhizobia and bradyrhizobia has assessed the linkage between symbiotic functioning and bacterial IGS types resident in nodules and/or used for determining rhizobial biodiversity. Quantifying N_2 _fixation in legumes and linking amounts of N-fixed to the IGS types found in their root nodules, could provide some indication of the symbiotic efficiency of resident bacterial populations used for establishing rhizobial biodiversity. That way, studies of legume agronomy in the context of N contribution could add value to bacterial biodiversity and phylogeny in relation to symbiotic functioning.

In this study, 9 cowpea genotypes were planted in field experiments in Botswana, South Africa and Ghana with the aim of i) trapping indigenous cowpea rhizobia in the 3 countries for isolation and molecular characterisation, ii) quantifying N-fixed in the cowpea genotypes using the ^15^N natural abundance technique, and iii) relating the levels of nodule functioning (i.e. N-fixed) to the IGS types found inside cowpea nodules, in order to assess strain IGS type symbiotic efficiency.

## Methods

### Experimental site descriptions

In Ghana, the experiments were conducted at the Savanna Agricultural Research Institute (SARI) site at Dokpong, Wa, in 2005. The site is located in the Guinea savanna, (latitude 10° 03' N, longitude 2° 30' W, and altitude 370 m) and has a unimodal rainfall (1100 mm annual mean) that starts in May and ends in September/October. The soils are classified as Ferric Luvisols [10]. Prior to experimentation, the site had been fallowed for 3 years. In South Africa, the Agricultural Research Council (ARC-Grain Crop Research Institute) farm at Taung, Potchefstroom, was used for the field trials. The Taung experimental site is located between latitudes 27° 30' S and longitudes 24° 30' E, and is situated in the grassland savanna with a unimodal rainfall (1061 mm annual mean) that begins in October and lasts until June/July the following year. The soil at Taung is structureless, and freely drained with a reddish-yellow colour. The site was cropped to maize (*Zea mays *L.) the previous year with the application of NPK fertiliser. In Botswana, the experimental site was located at Glenvalley near Gaborone, in the Botswana College of Agriculture in 2006. The farm is situated between 24° 40' S and 26° 09' E at an altitude of 1015 m and it is part of an open savanna agro-ecology with a unimodal rainfall (429 mm annual mean). The soil is classified as Ferric Luvisol [10] or Kanhaplic Haplustalf (Soil Taxonomy), and had not been cultivated before.

### Planting, harvesting and processing

Nine cowpea genotypes were used in this study, namely Omondaw, Brown eye, ITH98-46, IT82D-889, Apagbaala, Bechuana white, Glenda, Mamlaka and Fahari. Of these, Omondaw, Apagbaala (both farmer varieties) and Brown eye (an inbred cultivar) originated from Ghana; Mamlaka and Fahari (two farmer varieties) came from Tanzania; Glenda and Bechuana white were two improved commercial varieties originating from South Africa and Botswana respectively, while ITH98-46 and IT82D-889 were breeder varieties that came from IITA in Nigeria. The 9 cowpea genotypes were planted at Dokpong, Taung and Glenvalley in Ghana, South Africa and Botswana respectively, using a randomized complete block design with four replicate plots. Planting was done in mid-July in Ghana, early January in Botswana, and mid-October in South Africa, in accordance with the rainfall pattern of each country.

Plants were sampled from the inner part of the middle rows of each plot at 46 days after planting, and separated into shoots and nodules, in the case of Ghana and South Africa. The shoots were oven-dried at 60°C to constant weight for dry matter determination. Nodules were dried at 45°C and stored prior to DNA extraction. For the Botswana trial, only root nodules were sampled due to a sudden incidence of disease (cowpea rust). As a result, only the shoots from the Ghana and South Africa were milled to fine powder (0.85 mm sieve) for ^15^N analysis.

### ^15^N/^14^N isotopic analysis

About 2.0 mg of each milled sample was weighed into a tin capsule (Elemental Microanalysis Ltd, Okehampton, UK) and run on a Thermo Finnigan Delta Plus XP stable light isotope mass spectrometer (Fisons Instrument SpA, Strada Rivolta, Italy) coupled via a Conflo III device to Thermo 1112 Flash elemental analyzer against an internal reference plant material (*Nasturtium *sp.) The *Nasturtium *sp. had been calibrated against an IAEA standard (Air for N) and the results expressed relative to air.

The isotopic composition of ^15^N was measured as the difference in the number of atoms of ^15^N to ^14^N in atmospheric (atm) N_2 _[11,12]:

Whole-plant ^15^N natural abundance was calculated as an average of the ^15^N natural abundance values of all plant parts weighted by their respective total N [13]:

The N content of each organ was determined as the product of %N and sample weight [14]:

### %Ndfa and N-fixed values

The proportion of N derived from biological N_2 _fixation in the plant was calculated as [15]:

Where δ^15^N_ref _is the mean ^15^N natural abundance of non-N_2_-fixing reference plant shoots, δ^15^N_leg _is the mean ^15^N natural abundance of the legume (cowpea) shoots, and the B value is the ^15^N natural abundance of cowpea shoots which were dependent solely on symbiotic N_2 _fixation for their N nutrition. B value of -1.759 for cowpea shoots was used in calculating %Ndfa [3]. At Wa, sorghum and maize crops planted at the same time and growing on an adjacent field (as monocrops) were used as reference plants; they had an average δ^15^N value of +7.12‰. For Taung, an *Eragrostis *sp. and an unidentified herbaceous weed growing in the field with cowpea were analysed as the reference plants. Their average δ^15^N value of +5.03‰ was used to estimate %Ndfa in cowpea. While the cowpea plants were raised on ridges, the *Eragrostis *sp. and the herbaceous weed sampled as reference plants, were growing on the ploughed unridged area around the experimental plots. The amount of N-fixed was calculated as [16]:

The amount of N-fixed in each cowpea shoot was divided by the plant's nodule mass and age to obtain the specific nodule activity, expressed as μg N - fixed.mg nod DM^-1^.d^-1 ^[17].

### Nodule harvest and DNA extraction

Two hundred and seventy (270) nodules were harvested from the 9 cowpea genotypes planted in Ghana, South Africa and Botswana for DNA extraction. The nodules harvested were generally representative of the total nodule pool per plant, and were all effective in N_2 _fixation based on the pink internal colour (i.e. presence of leghaemoglobin). Total DNA (plant and microbial) was extracted from each of the 270 nodules, using the method described by [18]. To sterilise the nodules, they were rehydrated in sterile distilled water, immersed in 3.3% w/v Ca(OCl)_2 _for 3 min, rinsed in sterile water, followed by soaking in 96% ethanol and rinsed twice in sterile distilled water. Each nodule (about 4 mg in weight) was crushed in 100 μL TES/sucrose buffer (20 mM Tris-HCl, pH 8.0, 50 mM EDTA di-sodium, pH 8.0, 8% p/v) in a sterilised 1.5 mL Eppendorf tube (using a plastic pestle sterilised in absolute ethanol). Lyzozyme (4 mg/μL) was added to the crushed nodule macerate, vortexed for 20 s and incubated at 37°C for 15 min. A solution of GES (0.05 mM guanidine thiocyanate, 0.1 M EDTA di-sodium, pH 8.0, 1% N-Lauroylsarcosine sodium salt) was added to the lysed nodule homogenate, vortexed again for 20 s and incubated at 65°C for 15 min. The GES-cell lysate mixture was centrifuged at 10000 × g in a 3K15 Model Sigma centrifuge for 15 min at 4°C and the supernatant transferred into a new tube. Total DNA was pelleted by centrifuging at 4°C at 10000 × g for 15 min. The supernatant was discarded, and 0.5 mL 95% ethanol added to the pellet and centrifuged again at 4°C at 10000 × g for 15 min. This was repeated twice. The DNA pellet was then air-dried, re-dissolved in 50 to 250 μL of 8 mM NaOH and the pH adjusted to pH 7.5 by adding 8 μL of 0.1 M HEPES (N-2-Hydroethylpiperazine-N'-2-ethanesulfonic acid) for every 50 μL of the NaOH used to dissolve DNA. The purity and quantity of the DNA was controlled by horizontal electrophoresis in 0.8% Sigma II agarose gel, using a molecular weight marker (Smart Ladder) for gel calibration. Electrophoresis was performed at 100 V for 30 min. The gel was stained in an aqueous solution of ethidium bromide (1 μg/mL) for 30 min, rinsed with sterile distilled water for 15 min and photographed under UV light with Gel Doc (Bio-Rad) software.

### PCR amplification and restriction fragment analysis

In this study, we chose PCR-RFLP and sequencing of the IGS region because of its great resolution power with symbiotic rhizobia [19] and the fact that the region provides taxonomic information similar to that obtained by DNA-DNA hybridisation [20]. Depending on its concentration and the amount of impurities present, each DNA sample was diluted with sterile MilliQ water and PCR performed in a Perkin Elmer 2400 Thermal cycler in a total volume of 25 μL reaction mixture using Ready-to-go Taq DNA polymerase (Pharmacia Biotech). A negative control with water (no DNA) was included in all the PCR runs. The 16S-23S rDNA PCR amplification was carried out using two primers, FGPL132-38 and FGPS1490-72 (Table 1). The protocol used included initial denaturation at 94°C for 15 min; 35 cycles of denaturation (30 s at 94°C), annealing (30 s at 55°C), extension (72°C for 1 min) and final extension at 72°C for 7 min. Amplified DNA products were separated by horizontal gel electrophoresis in 0.8% agarose gel. RFLP was carried out using a total volume of 20 μL containing 8 or 10 μL PCR products (depending on the intensity of the band on the PCR control gel), 1 μL endonuclease, 2 μL of the relevant buffer and 9 or 7 μL of ultrapure water (depending on the volume of the PCR products used). HaeIII and MspI restriction enzymes were used. The mixture was incubated at 37°C overnight. Restricted DNA fragments were analyzed after migration in 3% agarose gel at 80 V for 90 min. Electrophoregrams with similar migratory patterns were grouped together and assigned to the different IGS groups (IGS types I to XVIII).

**Table 1:** Primers used for PCR and sequencing reactions

Primer	Primer sequence (5'-3')	Target gene	Reference
FGPL 132-38	5'-CCGGGTTTCCCCATTCGG-3'	IGS rDNA	[28]
FGPS 1490-72	5'-TGCGGCTGGATCCCCTCCTT-3'	IGS rDNA	[29]
BRIIe	5'-GGCTTGTAGCTCAGTTGGTTAG-3'	IGS rDNA	COGENICS, France
BR4r	5'-CGAACCGACCTCATGC-3'	IGS rDNA	COGENICS, France

### Gene sequencing

One sample per group was selected for sequencing the 16S - 23S rDNA IGS gene. Prior to sequencing, the PCR products of the test samples were purified using QIAquick purification kit (Qiagen) and the sequencing done using four primers, FGPS1490-72, FGPL132-38, BRIIe and BR4r (COGENICS, Meylan, France, see Table 1). The sequences were analyzed from electrophoregrams and corrected using 4Peaks software (2005 Mek and Tsj.com, Netherlands). The parts of sequences corresponding to 16S and 23S rDNA genes were subtracted to obtain single IGS sequences which were aligned with CLUSTALX [21] and the closely related sequences were included in following analyses. Phylogenetic analysis was done using the CLUSTALX and phylogenetic trees constructed using the neighbour-joining method [22]. A bootstrap confidence analysis was performed on 1000 replicates to determine the reliability of the distance-tree topology obtained [23]. Graphic representation of the resulting trees was done using NJPLOT software [24].

## Results

### Plant growth and symbiotic performance of 9 cowpea genotypes

Analysis of data on nodule numbers, nodule mass, shoot dry matter and grain yield using One-Way ANOVA revealed significant differences between and among the 9 cowpea genotypes (Tables 2 and 3). At Wa, for example, Bechuana white and IT82D-889 produced the highest nodule number per plant while Brown eye and Apagbaala showed the least (Table 2). At Taung in South Africa, Fahari exhibited the highest nodulation with Brown eye again showing the least nodulation together with Omondaw (Table 3). Interestingly, IT82D-889 (which had the highest nodulation at Wa) also produced significantly the most nodule mass at Wa, with Mamlaka and Fahari producing very low nodule dry matter, followed by Brown eye and Fahari (Table 2). At Taung, IT82D-889 produced the largest nodule dry mass, followed by Bechuana white, while Mamlaka and Apagbaala showed the least nodule dry mass, even though they were intermediate in nodulation (Table 3).

**Table 2:** Symbiotic performance, dry matter and grain yield of 9 cowpea varieties grown in Wa, Ghana.

Genotype	Nodule number	Nodule DM	Shoot DM	δ^15^N	Ndfa
	per plant	mg.plant^-1^	g.plant^-1^	‰	%
Omondaw	35.0 ± 0.3b	1200.0 ± 57.7c	25.9 ± 3.7ab	-0.57 ± 0.2e	86.6 ± 0.1a
Brown eye	15.4 ± 0.3d	366.7 ± 33.3d	13.5 ± 1.6cd	0.30 ± 0.1d	76.8 ± 1.6c
Apagbaala	16.5 ± 1.4d	466.7 ± 33.3d	25.7 ± 2.8ab	0.76 ± 0.1bc	71.6 ± 1.3de
IT82D-889	41.3 ± 0.3a	2666.7 ± 66.7a	18.9 ± 1.4bc	-0.21 ± 0.1de	82.6 ± 1.6b
ITH98-46	26.6 ± 1.2c	500.0 ± 0.0d	8.8 ± 0.3d	0.50 ± 0.0cd	74.6 ± 0.2cd
Bechuana white	43.0 ± 0.8a	1733.3 ± 33.3b	18.7 ± 4.0bc	0.76 ± 0.1bc	71.6 ± 0.6de
Glenda	34.0 ± 1.4b	1733.3 ± 88.2b	27.7 ± 2.3a	0.81 ± 0.1a	70.7 ± 0.3e
Mamlaka	34.3 ± 1.5b	100.0 ± 11.0e	12.6 ± 2.0cd	1.00 ± 0.1a	69.3 ± 0.8e
Fahari	36.0 ± 0.8b	100.0 ± 10.0e	16.9 ± 1.2c	0.96 ± 0.2a	69.9 ± 1.8e
F-statistics	97.5***	384***	7.4***	29.4***	29.4***
					
	**N content**	**Grain yield**	**N-fixed**		
	**mg.plant^-1^**	**kg.ha^-1^**	**mg.plant^-1^**	**kg.ha^-1^**	

Omondaw	1077.5 ± 130.2ab	791.2 ± 144.8a	933.8 ± 111.8a	155.6 ± 18.6a	
Brown eye	705.5 ± 97.0cd	865.6 ± 93.8a	540.0 ± 68.2bcd	90.0 ± 11.4bcd	
Apagbaala	1233.4 ± 164.8a	723.1 ± 228.1a	887.6 ± 134.4a	147.9 ± 22.4a	
IT82D-889	896.1 ± 50.1abc	687.6 ± 104.3a	738.7 ± 29.5ab	123.1 ± 4.9ab	
ITH98-46	392.8 ± 9.1d	862.3 ± 59.5a	292.9 ± 6.7d	48.8 ± 1.1d	
Bechuana white	837.3 ± 171.1bc	652.7 ± 76.7a	599.9 ± 124.2bc	100.0 ± 20.7bc	
Glenda	1244.3 ± 111.9a	888.1 ± 102.6a	879.3 ± 79.3a	146.5 ± 13.2a	
Mamlaka	579.5 ± 94.9cd	686.7 ± 47.6a	401.0 ± 63.0cd	66.8 ± 10.5cd	
Fahari	727.5 ± 54.1bcd	252.5 ± 62.4b	507.3 ± 28.7bcd	84.5 ± 4.8bcd	
F-statistics	7***	3**	7.6***	8***	

Values (Mean ± SE) with dissimilar letters in a column are significantly different at p ≤ 0.001 (***); p ≤ 0.01 (**)

**Table 3:** Symbiotic performance, dry matter and grain yield of 9 cowpea varieties grown at Taung, South Africa.

Genotype	Nodule number	Nodule DM	Shoot DM	δ^15^N	Ndfa
	per plant	mg.plant^-1^	g.plant^-1^	‰	%
Omondaw	15.6 ± 1.2d	236.7 ± 14.4de	11.4 ± 1.4ef	-0.2 ± 0.0de	77.0 ± 0.6bcd
Brown eye	15.8 ± 2.4d	361.7 ± 19.5cde	12.3 ± 1.7def	0.2 ± 0.0c	72.6 ± 1.0cd
Apagbaala	24.1 ± 0.6c	131.7 ± 10.1e	12.1 ± 0.7def	0.9 ± 0.1b	61.2 ± 2.0ef
IT82D-889	20.3 ± 0.3cd	1437.2 ± 117.9a	13.5 ± 0.6cde	0.9 ± 0.1b	92.9 ± 1.7a
ITH98-46	22.8 ± 2.8c	263.3 ± 8.8de	7.4 ± 0.9f	-0.5 ± 0.1ef	81.5 ± 1.3bc
Bechuana white	33.4 ± 0.5b	665.3 ± 71.8b	18.1 ± 2.0bc	0.1 ± 0.0cd	85.4 ± 6.1ab
Glenda	33.4 ± 0.5b	398.9 ± 7.3cd	22.2 ± 0.8b	1.9 ± 0.3a	59.3 ± 3.6f
Mamlaka	24.5 ± 1.4c	132.2 ± 15.4e	16.7 ± 2.9cd	0.7 ± 0.1b	69.8 ± 4.9d
Fahari	42.5 ± 0.6a	538.6 ± 6.1bc	27.8 ± 1.9a	-0.6 ± 0.0f	77.0 ± 0.6bcd
F-statistics	31.1***	27.6***	15.1***	44.3***	10.5***
					
	**N content**	**Grain yield**	**N-fixed**		
	**mg.plant^-1^**	**kg.ha^-1^**	**mg.plant^-1^**	**kg.ha^-1^**	

Omondaw	580.6 ± 88.9cde	2231.3 ± 297.9a	446.3 ± 46.2bcd	74.4 ± 7.0bcd	
Brown eye	563.1 ± 74.0cde	512.1 ± 66.1c	409.6 ± 57.5bcd	68.3 ± 9.6bcd	
Apagbaala	566.2 ± 58.8cde	579.8 ± 47.7c	348.0 ± 47.5cd	58.0 ± 7.9cd	
IT82D-889	473.1 ± 15.2de	1427.7 ± 145.0b	438.9 ± 6.9bcd	73.1 ± 1.1bcd	
ITH98-46	378.9 ± 35.5e	1500.4 ± 167.6b	307.7 ± 38.3d	51.3 ± 6.4d	
Bechuana white	727.5 ± 84.2cd	1494.3 ± 115.4b	620.8 ± 47.5b	103.5 ± 13.7b	
Glenda	1021.0 ± 99.3ab	1892.1 ± 129.9ab	598.8 ± 22.1b	99.8 ± 3.7b	
Mamlaka	784.8 ± 39.1bc	1651.8 ± 96.2ab	561.4 ± 40.6bc	93.6 ± 8.4bc	
Fahari	1219.3 ± 90.3a	1588.2 ± 107.7b	931.6 ± 27.3a	155.3 ± 4.5a	
F-statistics	10.1***	8.8**	8.2***	8.2***	

At Wa, Omondaw and Glenda, which were second highest in nodulation, produced the largest shoot biomass and the highest amount of N-fixed compared to Mamlaka and Fahari (which had very low nodule mass). As with IT82D-889 and Brown eye, Omondaw and Glenda also produced the lowest amount of N-fixed and the least shoot biomass (Table 2). At Taung in South Africa, Fahari (which had the highest nodule number and was second in nodule mass) produced significantly the highest amount of N-fixed and the largest amount of shoot biomass (Table 3). In the same manner, Apagbaala, which had the least nodule mass showed (together with ITH98-46 and Omondaw) the least shoot biomass and the lowest amount of N-fixed (Table 3).

### Nodule occupancy

From the PCR-RFLP analysis, the IGS types of strains resident in 30 root nodules from each of the 9 cowpea genotypes were determined and percent nodule occupancy estimated. In total, 18 IGS types were found after analysing 270 nodules from 9 cowpea genotypes (Table 4). IGS type I was found in the nodules of only Omondaw, type II in both Omondaw and Bechuana white, type III in all the genotypes except Omondaw and Bechuana white, type IV in IT82D-889 only, type V in all genotypes except Omondaw, type VI in Glenda, Brown eye and Fahari, type VII in Omondaw, IT82D-889, Bechuana white and Glenda, type VIII in all the genotypes except Glenda, types IX, X, XI and XII in only Glenda, type XIII in only Fahari and Apagbaala, type XIV in only Apagbaala, types XV, XVI and XVII in only Fahari, and type XVIII in only Apagbaala (Table 4). Nodules from Fahari contained the highest number (8) of IGS types, followed by Apagbaala with 6, IT82D-889 with 5, Omondaw, Bechuana white and Brown eye each with 4, and ITH98-46 and Mamlaka each with 3 IGS types (Table 4).

**Table 4:** Percent nodule occupancy by different IGS types in 9 cowpea genotypes grown in Ghana, Botswana and South Africa

	Percent nodule occupancy per cowpea variety								
IGS Type	Omondaw	IT82D-889	Bechuana white	Glenda	ITH98-46	Brown eye	Mamlaka	Fahari	Apagbaala
I	33.3	0	0	0	0	0	0	0	0
II	44.4	0	15.8	0	0	0	0	0	0
III	0	28	0	16	68.2	83.3	15.8	13.3	28.6
IV	0	11	0	0	0	0	0	0	0
V	0	25	57.9	36	26.3	16.7	5.3	6.7	28.6
VI	0	0	0	8	0	0	0	6.7	0
VII	11.1	4	10.5	4	0	0	0	0	0
VIII	11.2	32	15.8	0	5.5	0	78.9	46.6	16.6
IX	0	0	0	16	0	0	0	0	0
X	0	0	0	4	0	0	0	0	0
XI	0	0	0	4	0	0	0	0	0
XII	0	0	0	4	0	0	0	0	0
XIII	0	0	0	0	0	0	0	13.3	16.6
XIV	0	0	0	0	0	0	0	0	4.8
XV	0	0	0	0	0	0	0	6.7	0
XVI	0	0	0	0	0	0	0	6.7	0
XVII	0	0	0	8	0	0	0	0	0
XVIII	0	0	0	0	0	0	0	0	4.8

Values (Mean ± SE) with dissimilar letters in a column are statistically significant at p ≤ 0.001 (***); p ≤ 0.01 (**)

The per-country data for nodule occupancy by each strain (or IGS type) are shown in Table 5. IGS types I, IV, IX, X, XI, XIII, XIV, XVI, XVII and XVIII were only found in the root nodules of cowpea plants grown at Taung, South Africa (but not in those from Ghana and Botswana), while XV and XIX were exclusively found in nodules from Glenvalley in Botswana, and IGS type XII was unique to nodules from Ghana.

**Table 5:** Percent nodule occupancy by different IGS types per country

PCR-RFLP IGS type	Sample no. of IGS types selected for gene sequencing	Percent nodule occupancy per country		
		South Africa	Botswana	Ghana
I	5	100	0	0
II	8	25	0	75
III	116	71.4	18.6	0
IV	22	100	0	0
V	68	78.6	9.4	12
VI	103	85.7	14.3	0
VII	27	60	0	40
VIII	36	94.2	0	5.8
IX	104	100	0	0
X	115	100	0	0
XI	117	100	0	0
XII	201	0	0	100
XIII	91	100	0	0
XIV	106	100	0	0
XV	7/116	0	100	0
XVI	146	100	0	0
XVII	150	100	0	0
XVIII	153	100	0	0

### Strain IGS type diversity from PCR-RFLP analysis

**Figure 1:**
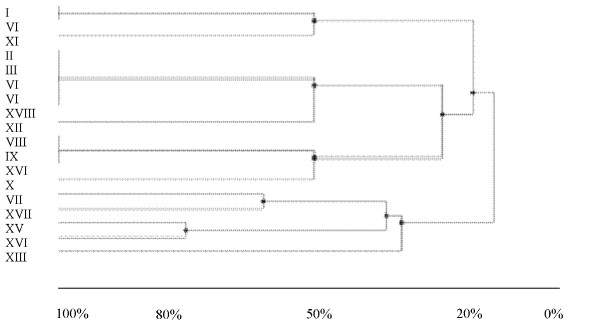
**UPGMA dendrogram derived from PCR-RFLP of bradyrhizobial DNA in cowpea nodules collected from South Africa, Botswana and Ghana, generated by HaeIII digestion of amplified rDNA products**. Scale indicates % similarity.

When DNA from each nodule was amplified with the two primers, FGPL 132-38 and FGPS 1490-72, a PCR product of about 900 bp was found that corresponded to the size of 16S-23S IGS region. A comparison of *Bradyrhizobium *DNA extracted from root nodules of the 9 cowpea genotypes using IGS PCR-RFLP analysis with HaeIII and Msp restriction enzymes yielded a UPGMA dendogram from HaeIII, which showed 4 divergent lineage groups for the eighteen IGS types. Four clusters were discernible at 50% similarity level using HaeIII (Figure 1). Cluster 1 consisted of bacterial DNA from nodules of Omondaw (grown in South Africa and Ghana), IT82D-889 and Bechuana white (grown in South Africa), and Glenda (grown in Ghana). Cluster 2, on the other hand, was made up of i) IGS types from nodules of all the 9 genotypes grown in South Africa, ii) IGS types from nodules of ITH98-46, IT82D-889, Glenda, Mamlaka, Brown eye, Bechuana white and Apagbaala grown in Botswana, and iii) IGS types from nodules of Glenda, Bechuana white and IT82D-889 grown in Ghana. In contrast, cluster 3 consisted of IGS types coming from root nodules of only Glenda and Fahari grown in South Africa. Like cluster 2, cluster 4 was made of IGS types from nodules of cowpea genotypes grown in all the 3 countries.

### Strain IGS type symbiotic efficiency

**Figure 2:**
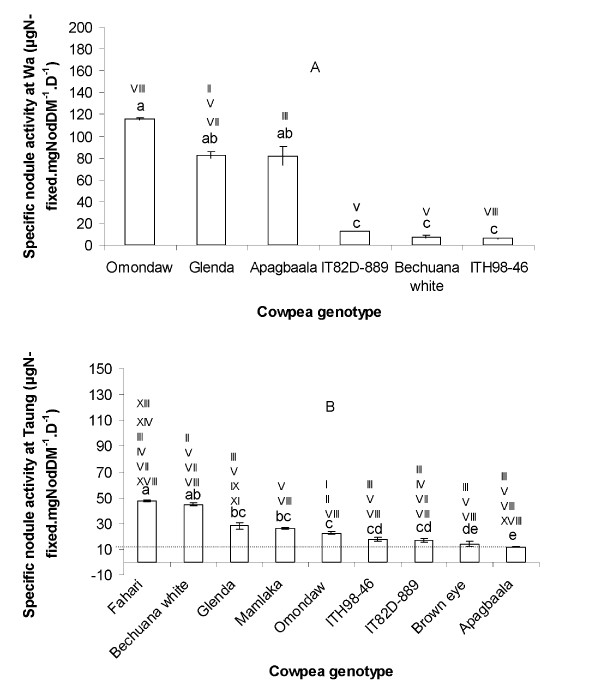
**Specific nodule activity for the 9 genotypes grown at A) Wa in Ghana, B) Taung in South Africa**. Bars with dissimilar letters indicate significant differences at p ≤ 0.05. Numerals on the top of each bar represent the different IGS types (strains) that were found in the cowpea nodules from the particular genotype.

Relating symbiotic functioning (measured here as specific nodule nitrogenase activity) to the IGS types found inside root nodules revealed significant differences in the N_2_-fixing efficiency of these IGS types (Figure 2). For example, IGS types V and VIII fixed very low N in IT82D-889 and Bechuana white relative to IGS type III in Apagbaala at Wa in Ghana (see Figure 2). It was also interesting to note that sole nodule occupancy by IGS type VIII in Omondaw resulted in significantly very high N yield relative to its poor performance as a sole occupant of nodules in ITH98-46 at Wa in Ghana (Figure 2A). Similar differences in symbiotic functioning were obtained for combinations of resident IGS types found in root nodules of the 9 cowpea genotypes at Taung in South Africa (Figure 2B).

### 16S-23S rDNA IGS sequencing

**Figure 3:**
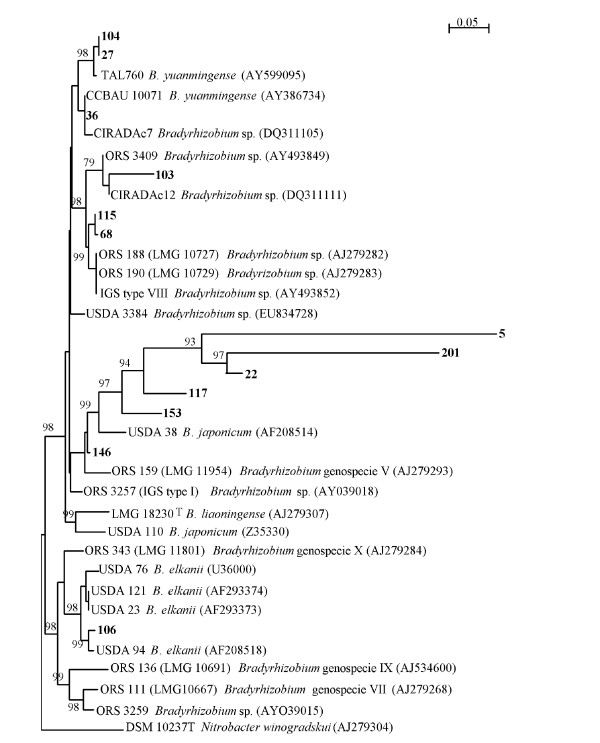
**Phylogenetic relationship among 16S-23S rDNA IGS types of from cowpea nodules, reference strains and more closed isolates based upon aligned 16S-23S rDNA IGS region sequences constructed as rooted tree using neighbour-joining method**. The bootstrap values (expressed as percentage of 1000 replications) shown at nodes are those greater than 70%.

Out of 18 IGS types samples submitted for gene sequencing (see Table 5), only 13 (i.e. samples with sequence numbers 104, 27, 36, 103, 115, 68, 5, 201, 22, 117, 153, 146 and 107) were successfully sequenced. As a result, the 13 16S-23S rDNA IGS sequences for *Bradyrhizobium *(i.e. sequence 104, 27, 36, 103, 115, 68, 5, 201, 22, 117, 153, 146 and 107) were deposited in the GenBank database under accession numbers [GenBank: FJ983128 to FJ983140] for sequence alignments with those of existing *Bradyrhizobium *species in the GenBank. The results from the Genbank database showed that IGS sequences 104, 27, 36, 115, 68 and 103 clustered with *Bradyrhizobium yuanmingense *and *Bradyrhizobium *sp. ORS 188, ORS 190 and USDA 3384 (Figure 3). Sequences 104, 27 and 36 showed little variation with *B. yuanmingense *(98% similarity), while IGS sequence 103 showed a 79% similarity with *Bradyrhizobium *sp ORS 3409 and CIRADAc12. IGS sequences 115 and 68 were found to be similar to *Bradyrhizobium *species ORS 188, ORS 190 and *Bradyrhizobium *genospecies VIII of [20]. Another cluster was formed by IGS sequences 5, 201, 22, 117, 153 and 146 around *Bradyrhizobium japonicum *USDA 38, *Bradyrhizobium *genospecies V of [20] and *Bradyrhizobium liaoningense*. The third cluster was made up of IGS sequence 106 with *B. elkani*, with the two having 98 - 99% similarities (Figure 3). The root-nodule bacteria nodulating cowpea in this study all belonged to the genus *Bradyrhizobium*.

## Discussion

Field measurements of N_2 _fixation using the ^15^N natural abundance revealed significant differences in plant growth and symbiotic performance of the 9 cowpea genotypes tested in South Africa and Ghana (Tables 2 and 3). The marked variation in plant growth (measured as dry matter yield) was linked to differences in overall nodule functioning. At Wa, for example, Omondaw and Glenda, which were among the highest in nodulation (nodule number and mass), showed the lowest ∂^15^N values, the highest %Ndfa, the highest amount of N-fixed, and thus produced the largest amount of plant growth and dry matter (Table 2). This was in contrast to Mamlaka and Fahari, which exhibited low nodulation and low N-fixed, and therefore produced the least shoot biomass at Wa (Table 2). At Taung in South Africa, Fahari which showed the best nodulation and the highest amount of N-fixed, recorded the highest amount of shoot biomass relative to Apagbaala, which exhibited the least nodulation, lowest amount of N-fixed, and thus produced the smallest plant biomass (Table 3). Of the 9 cowpea genotypes planted at Wa, Apagbaala was among the top 3 genotypes in N_2 _fixation (Table 2) due to its high specific nodule activity (Figure 2A). Yet in South Africa, Apagbaala and Omondaw were among the least in N_2 _fixation, even though they were the highest fixers in Ghana.

The better symbiotic performance of genotypes at one location (e.g. Omondaw and Apagbaala at Wa in Ghana) and their poor performance at another site (e.g. Taung in South Africa) could be attributed to the quality of nodule occupants (i.e. the resident IGS types inside root nodules, see Tables 4 and 5). As shown in Figure 2, when nodule functioning was related to nodule occupants, differences in N_2_-fixing efficiency were found among the resident IGS types, especially where there were clear cases of sole occupancy. For example, IGS types V and VIII fixed very low N in IT82D-889 and Bechuana white relative to IGS type III in Apagbaala (Figure 2A). It was also interesting to note that sole nodule occupancy by IGS type VIII in Omondaw at Wa resulted in significantly very high symbiotic N yield relative to its poor performance as a sole occupant of root nodules in ITH98-46 (Figure 2A). Similar differences in N_2_-fixing efficiency were found for combinations of IGS types resident in nodules of the 9 cowpea genotypes planted at Taung in South Africa (Figure 2B). However, at Taung, the nodules of the 9 cowpea genotypes were associated with very diverse and different IGS types, thus making assessment of individual IGS type symbiotic efficiency very difficult (Figure 2B). Even where an IGS type proved to be symbiotically very efficient with a particular genotype (e.g. IGS type VIII on Omondaw at Wa, Ghana), it can become low in N yield when in combination with other IGS types in nodules of same genotype (e.g. IGS type VIII on Omondaw at Taung, South Africa). In that case, either the associated IGS types I and II were ineffective in N_2 _fixation, or their co-occupancy in root nodules had a negative effect on the symbiotic efficiency of IGS type VIII (which as a sole occupant showed high N_2_-fixing efficiency). Although it has been demonstrated that the symbiotic performance of a double strain inoculant of *Rhizobium leguminosarum *was 2.5 times superior to their sole counterparts in subterranean clover [25], it is unclear whether the IGS types of those strains were the same or different. We therefore still do not know much about the negative or positive effects of IGS types on nodule functioning, especially when they are present as sole or multiple occupants on the same host plant.

The data on nodule occupancy clearly show that there was greater *Bradyrhizobium *biodiversity in the soil at Taung in South Africa relative to Ghana and Botswana, with many more IGS types found only in South Africa (Table 5). Cowpea genotypes Fahari, Glenda and Apagbaala proved to be the most promiscuous across the 3 countries in terms of trapping more strain IGS types: 8 by Fahari, 8 by Glenda and 6 by Apagbaala (Table 4).

In addition to the marked strain diversity observed from data on nodule occupancy, PCR-RFLP analysis using HaeIII and Msp restriction enzymes showed four lineage groups for the 18 IGS types (Figure 1). Gene sequencing of the 16S-23S rDNA IGS region further revealed phylogenetic diversity among the *Bradyrhizobium *IGS types occupying nodules of the 9 cowpea genotypes grown in South Africa, Botswana and Ghana (Figure 3). The gene sequence numbers 104, 27, 36, 103, 115, 68, 5, 201, 22, 117, 153, 146 and 106, representing samples selected from the 18 IGS types and deposited in the Genbank database, clustered with different *Bradyrhizobium *species. As shown in Figure 3, even though sequence 104 was from Glenda grown in South Africa, it formed a common clade with sequence 27 from Omondaw and Glenda grown in Ghana, and together they clustered around *B. yuanmingense *and *Bradyrhizobium *sp. Similarly, sequence 115 isolated from Glenda in South Africa shared a common clade with sequence 68 from 8 of the 9 cowpea genotypes (except Omondaw) grown in all 3 countries, and clustered with *Bradyrhizobium *sp ORS 188, ORS 190 and USDA 3384, just as sequence 103 isolated from South Africa and Botswana with Glenda, Brown eye and Fahari as trap hosts clustered around *Bradyrhizobium *sp ORS 3409 and CIRADc12. Perhaps the most important finding from the phylogenetic aspect of this study is the fact that cluster 2 (consisting of sequences 5, 201, 22, 117, and 153) formed its own distinct group, suggesting that it is a new *Bradyrhizobium *species (Figure 3). What is also unique about this cluster is that all the sequences (i.e. 5, 22, 117, 153 and 146, except for 201) originated from South Africa, though isolated from different cowpea genotypes (see Tables 4 and 5), again underscoring the greater *Bradyrhizobium *biodiversity in South Africa. Sequence 106 was the only one related to the *B. elkanii *group (see cluster 3, Figure 3), and was isolated only from South Africa with Apagbaala as trap host (Tables 4 and 5).

Although some reports claim to have isolated both bradyrhizobia (slow-growing) and rhizobia (fast-growing) from root nodules of cowpea [2,26], a recent study [9] found only *Bradyrhizobium *species in the root nodules of cowpea grown in South Africa and Botswana. In contrast, the Chinese have identified both rhizobia and bradyrhizobia in cowpea nodules [8]. In this study, we also found only bradyrhizobial strains in cowpea nodules when bacterial DNA was analyzed directly from nodules of cowpea plants grown in Ghana, Botswana and South Africa (see Figure 3).

Taken together, the data from studies of nodule occupancy, PCR-RFLP analysis, IGS type symbiotic efficiency and gene sequencing indicate greater biodiversity of cowpea bradyryhizobia in Africa, especially in South Africa. This was evidenced by the different IGS types found in cowpea nodules, as well as the phylogenetically-diverse groups obtained from the Genbank database. The observed strain diversity associated with the 9 cowpea genotypes led to different levels of IGS type symbiotic efficacy in same hosts at different sites, and in different hosts at same experimental site (Figure 2). Thus, the differences in IGS type diversity and symbiotic efficiency could account for the genotype × environment interaction that made it difficult to select superior cowpea genotypes for use across Africa.

In this study, the origin of cowpea genotypes showed no specific trend in their ability to trap IGS types across the 3 countries. However, many IGS types appeared to have clustered along geographical lines (Figure 1); for example, cluster 2 consisted exclusively of IGS types isolated from soils in Southern Africa. A number of studies also found clustering of bradyrhizobial isolates according to geographical regions [9,6,27]. Although in another study [9] none of the isolates examined showed similarity with *B. japonicum *and *B. liaoningense *[9], sequence 146 in this study was closely related to *B. japonicum *USDA 38 (AF208514).

## Conclusion

We have shown here that i) cowpea is strongly dependent on N_2 _fixation for its N nutrition in South Africa, Ghana and Botswana, ii) the diversity of cowpea-nodulating bradyrhizobia was much higher in South Africa compared to Botswana and Ghana, iii) some strains from Southern Africa were phylogenetically very distinct, thus suggesting that they may be a new *Bradyrhizobium *species. Strain IGS type symbiotic efficiency was assessed for the first time in this study, and the data showed significant differences between and among the IGS types in terms of their symbiotic efficiency.

## Authors' contributions

FPM performed the PCR and RFLP and wrote the manuscript. AKB collected data from Ghana and South Africa, and did the isotopic analysis. TKW supervised the molecular work done by FPM and performed the sequence alignment. FDD is the PhD supervisor of FPM and AKB, he conceptualised the study and edited the manuscript before submission. All authors have read the manuscript before submission. All authors have read and approved the final manuscript.
